# The Impacts of Inclusion in Clinical Trials on Outcomes among Patients with Metastatic Breast Cancer (MBC)

**DOI:** 10.1371/journal.pone.0149432

**Published:** 2016-02-22

**Authors:** Ji Yun Lee, Sung Hee Lim, Min-Young Lee, Hae Su Kim, Jin Seok Ahn, Young-Hyuck Im, Yeon Hee Park

**Affiliations:** Department of Medicine, Samsung Medical Center, Sungkyunkwan University School of Medicine, Seoul, Korea; Taipei Medical University, TAIWAN

## Abstract

**Background:**

Metastatic breast cancer (MBC) remains a devastating and incurable disease. Over the past decade, the implementation of clinical trials both with and without molecular targeted therapeutics has impacted the daily clinical treatment of patients with MBC. In this study, we determine whether including MBC patients in clinical trials affects clinical outcomes.

**Methods:**

We retrospectively reviewed data for a total of 863 patients diagnosed with initial or recurrent (after receiving adjuvant systemic treatments following surgery) metastatic disease between January 2000 and December 2013. Data were obtained from the breast cancer database of Samsung Medical Center.

**Results:**

Among the 806 patients selected for inclusion, 188 (23%) had participated in clinical trials. A total of 185 clinical trials were conducted from 2000 to 2014. When compared with earlier periods (n = 10 for 2000–2004), clinical trial enrollment significantly increased over time (n = 103 for 2005–2009, *P* = 0.024; n = 110 for 2010–2014, *P* = 0.046). Multivariate analyses revealed that biologic subtype, distant recurrence free interval (DRFI), and clinical trial enrollment were independent predictors of overall survival. Patients who participated in clinical trials showed improved survival, with a hazard ratio of 0.75 (95% CI, 0.59–0.95), which was associated with a 25% reduction in the risk of death. However, subgroup analysis showed that this improved survival benefit was not maintained in patients with triple negative breast cancer (TNBC).

**Conclusions:**

Although not conclusive, we could speculate that there were differences in the use of newer agents or regimens over time, and these differences appear to be associated with improved survival.

## Introduction

Breast cancer is the most common female cancer and one of the leading causes of death among women worldwide [1, 2]. It is estimated that 30–50% of patients with early to locally advanced breast cancer at diagnosis experience relapse despite the use of adjuvant systemic treatment after surgery [3]. In addition, 5–10% of patients with breast cancer present with metastatic disease at diagnosis [4, 5]. Patients with metastatic disease at either initial diagnosis or relapse have traditionally been considered incurable with conventional treatment. However, over the past decade, the survival of patients with metastatic breast cancer (MBC) has improved slowly [6, 7]. Potential explanation for this improvement are early detection of metastatic disease [8], new drugs [9–11], advances in supportive care [12, 13], and palliative surgery or radiotherapy [14].

Over the past decade, a number of trials have demonstrated improved survival in patients with MBC when treatments with newer hormone agents or chemotherapeutic regimens were compared with previous standards [9, 15–18]. In particular, trastuzumab increases the clinical benefit of first-line chemotherapy in cases of MBC that overexpress human epidermal growth factor receptor (HER2) [9]. However, it is not clear whether the clinical outcomes of patients who participate in clinical trials differ from those of patients who receive conventional treatment without inclusion in clinical trials.

The number of clinical trials exploring treatments for MBC that is refractory to conventional treatment has increased rapidly in Korea since the year 2000. We explored the impact of inclusion in clinical trials on survival among patients with MBC, and sought to identify patients who were the most likely to benefit from clinical trials. We hypothesized that the outcomes of patients who participate in clinical trials are improved compared to those of patients who received only conventional treatments.

## Materials and Methods

### Study Design and Sample

**Fig 1:**
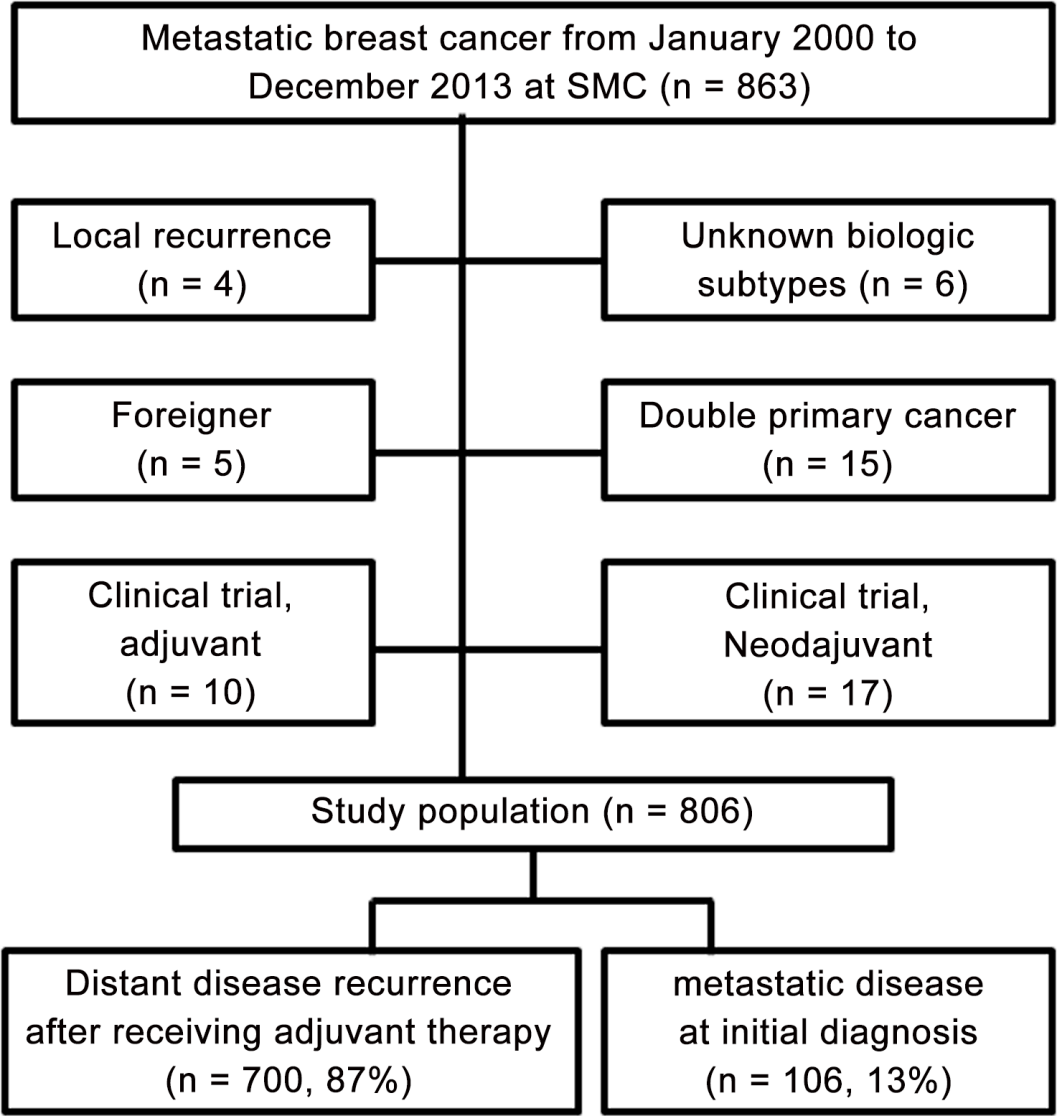
Summary of inclusion criteria.

We retrospectively reviewed data for a total of 863 patients with metastatic disease at either initial diagnosis or recurrence after receiving adjuvant therapy between January 2000 and December 2013. Data were obtained from the breast cancer database of Samsung Medical Center. We excluded patients with local and/or contralateral recurrence, patients with double primary cancer, and patients who participated in clinical trials in neoadjuvant and/or adjuvant settings. A total of 806 patients were included in this study (Fig 1). This study was performed in accordance with the Declaration of Helsinki and approved by Institutional Review Board of Samsung Medical Center. The patient records /information was anonymized and de-identified prior to analysis.

### Data collection

Clinical data were obtained by review of all patient medical records. Baseline patient characteristics collected for analysis included age, biological subtype according to the hormone receptor (HR) status, disease status, year of diagnosis with MBC, and medical treatment. Breast cancer is a heterogeneous disease that is classified according to biological subtype determined using immunohistochemistry (IHC) for estrogen receptor (ER), progesterone receptor (PgR), and human epidermal growth factor receptor type 2 (HER2). We stratified the patients into three groups: HR + (defined as ER+ and/or PgR+, HER2-), HER2+ (defined as HER2+ regardless of ER and/or PgR), and triple negative breast cancer (TNBC) (defined as lacking ER, PgR, and HER2 expression). Patients were divided by disease status at diagnosis with MBC: metastatic disease at initial diagnosis, distant recurrence-free interval (DRFI) < 5 years, and DRFI ≥ 5 years [6]. We also divided the patients into groups according to the time period of diagnosis with MBC: 2000 to 2004, 2005 to 2009, and 2010–2013. The data-cutoff date for the results presented here was December 16 2014. Given the retrospective nature of study, clinical data of performance status at diagnosis with MBC was limited to evaluate.

### Statistical analysis

Overall survival (OS) was defined as the interval from the date of diagnosis of distant metastasis to death from any cause. All patients who remained alive at the date of last analysis were censored. Patient characteristics were compared using chi-square and Fisher’s exact tests (categorical variables). Survival probabilities were estimated using the Kaplan-Meier method and compared for significant differences using log-rank analysis. Multivariate analyses were performed using the Cox proportional hazards regression model. All analyses were carried out using SPSS 20.0 (IBM SPSS Statistics, IBM Corp., Armonk, NY, USA) and statistical significance was set at *P* ≤ 0.05.

## Results

### Patient characteristics

Table 1 shows baseline patient characteristics according to biologic subtype. Of 806 total patients, 410 (51%) patients were HR+, 224 (28%) were HER2+, and 172 (21%) were TNBC. The median age at diagnosis with MBC was 48 years (range, 25–86 years) and distributions of age among the biological subtypes were similar. Patients with unfavorable biological subtypes including HER2+ and TNBC had shorter DRFI (*P* < 0.001) and were more likely to be treated with cytotoxic chemotherapy (*P =* 0.003) than patients with HR+. The various chemotherapies and hormone agents were received for the treatment of MBC by time periods of diagnosis with MBC (S1 Table). Proportion of patients received palliative chemotherapeutic agent or hormone therapy was similar over time periods. The anthracyclines and vinorelbine were used less commonly in the later time period, significantly (*P* < 0.001). Another trend was the increased use of eribulin for MBC in the later time period (*P =* 0.036). The proportions of time period of recurrence differed according to biologic subtype (*P* = 0.001).

**Table 1:** Characteristics according to biological subtype (n = 806).

Variable		Total	HR+	HER2+	TNBC	*P*
		n =	n = 410	n = 224	n = 172	value
		806	(51%)	(28%)	(21%)	
Mean age, year (SD)		49 (10)	50 (11)	49 (10)	49 (10)	0.744
Disease status at diagnosis with MBC	MBC at initial diagnosis	106 (13)	58 (14)	34 (15)	14 (8)	< 0.001
	DRFI < 5 years	548 (68)	237 (58)	165 (74)	146 (85)	
	DRFI ≥ 5 years	152 (19)	115 (28)	25 (11)	12 (7)	
Palliative cytotoxic chemotherapy	No	154 (19)	101 (25)	24 (11)	29 (17)	0.003
	Yes	652 (81)	309 (75)	200 (89)	143 (83)	
Clinical trial enrollment	No	618 (77)	313 (76)	159 (71)	146 (85)	0.106
	Yes	188 (23)	97 (24)	65 (29)	26 (15)	
Time period of diagnosis with MBC	2000–2004	152 (19)	64 (16)	51 (23)	37 (22)	0.001
	2005–2009	415 (51)	205 (50)	113 (50)	97 (56)	
	2010–2013	239 (30)	141 (34)	60 (27)	38 (22)	
No.of clinical trial enrollment (n = 188)	1	153 (81)	81 (84)	49 (75)	23 (89)	0.850
	2	31 (17)	14 (14)	14 (22)	3 (11)	
	3	4 (2)	2 (2)	2 (3)	0	
Biologics containing clinical trial (n = 188)	No	105 (56)	69 (71)	15 (23)	21 (81)	0.087
	Yes	83 (44)	28 (29)	50 (77)	5 (19)	

SD, standard deviation; MBC, metastatic breast cancer; DRFI, distant recurrence-free interval.

### Trends in clinical trials for patients with metastatic breast cancer

**Fig 2:**
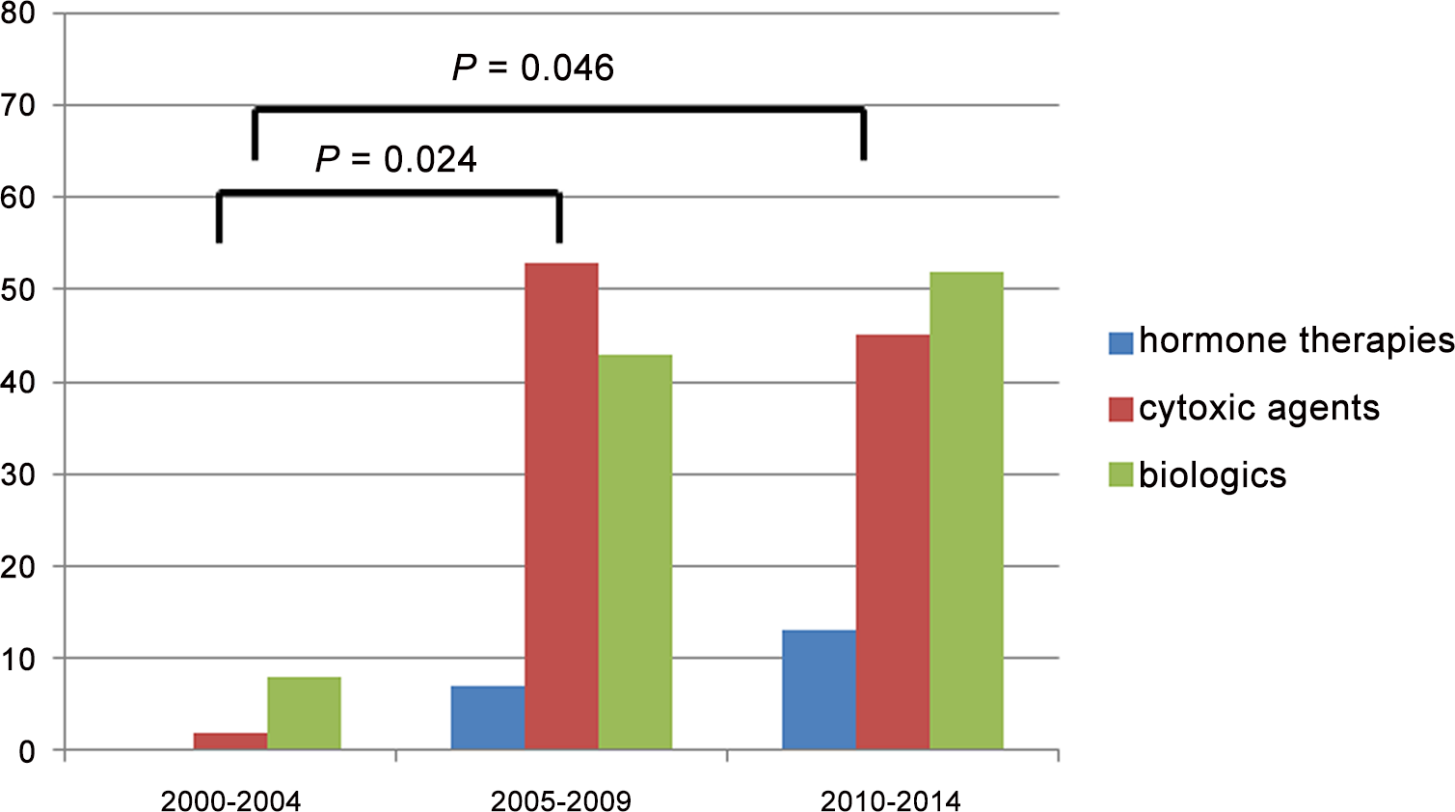
Trends of participating in clinical trials among patients with MBC from 2000 to 2014.

Fig 2 shows trends in clinical trials of treatments for MBC from 2000 to 2014. Among 806 patients, 188 (23%) had participated in clinical trials, and 223 clinical trials were conducted from 2000 to 2014 at SMC. When compared with earlier periods (n = 10 for 2000–2004), a significant increase in the number of patients who were enrolled on a clinical trial was observed over time (n = 103 for 2005–2009, *P* = 0.024; n = 110 for 2010–2014, *P* = 0.046). The clinical trials that were received for the treatment of MBC are outlined in S2 Table.

### Survival outcomes and prognostic factors

**Fig 3:**
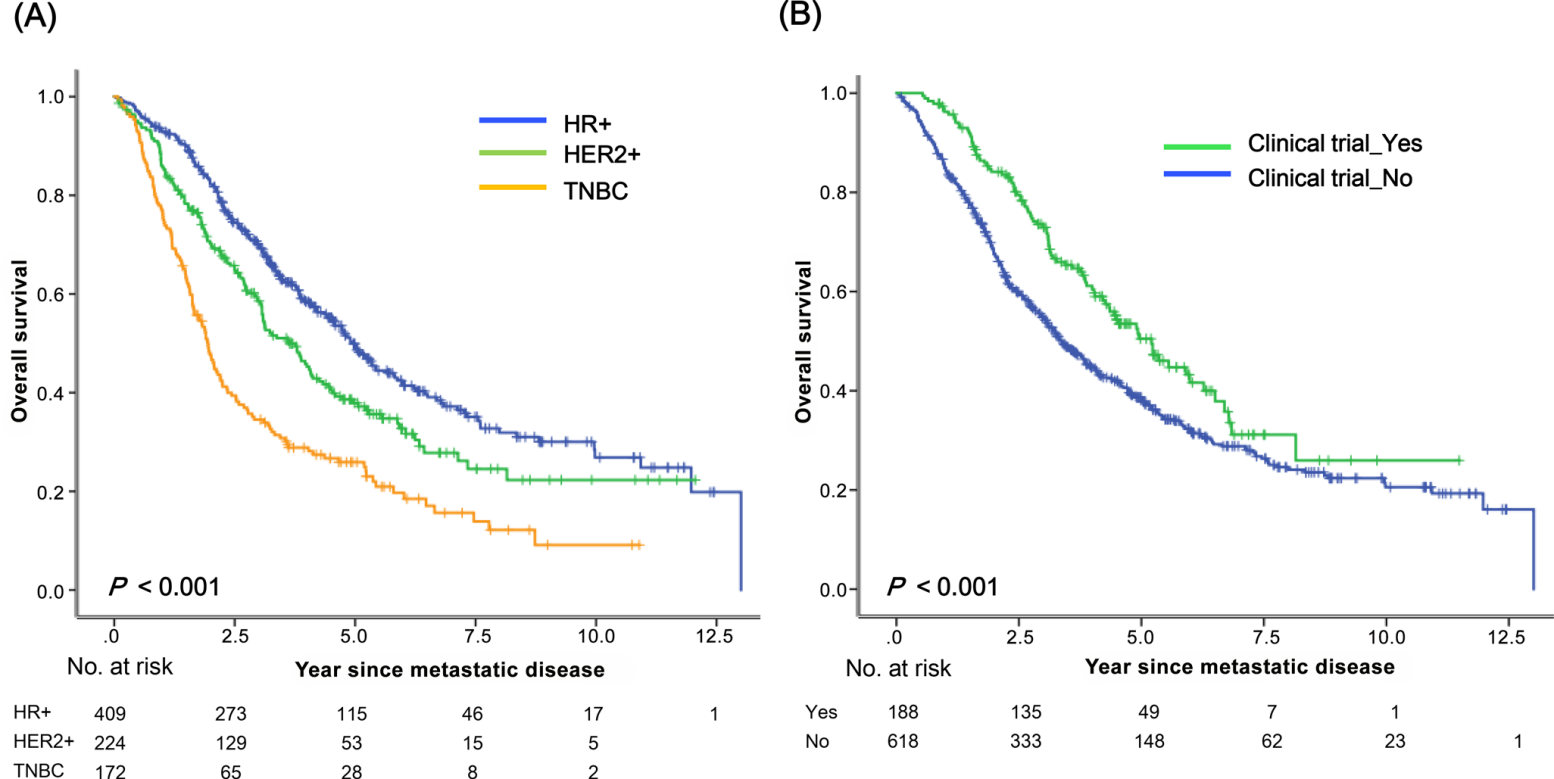
Survival after diagnosis with MBC according to (a) biological subtype and (b) clinical trial enrollment.

The median follow-up durations among all patients were 6.2 years (range, 0.9–15.4 years), 6.6 years (range, 0.5–14.5), and 6.9 years (range, 1.4–14.1) for HR+, HER2+, and TNBC, respectively. The median survival after diagnosis with MBC was 3.8 years (95% CI, 3.4–4.3 years). Estimated 5-year and 10-year survival rates were 41% and 22%, respectively. Biological subtype was strongly associated with survival after metastasis (*P* < 0.001) (Fig 3A). Estimated 5-year survival rates among patients with HR+, HER2+, and TNBC were 50%, 37%, and 25%, respectively. The estimated 5-year survival rate was significantly higher for patients who had enrolled in clinical trials than for patients who had not (50% vs. 38%, *P* < 0.001) (Fig 3B). Patients with a longer DRFI (≥ 5 years) showed significantly better survival outcomes than those with a shorter DRFI (< 5 years) (S1A Fig). There was a significant improvement in survival for women with MBC over time (S1B and S1C Fig).

Table 2 shows the hazards ratios (HRs) estimated using the Cox proportional hazards model. Biological subtype (HER2+ or TNBC), shorter DRFI (< 5 years), and lack of clinicial trial participation were associated with significantly shorter survival. Patients who were enrolled in clinical trials showed improved survival with a HR of 0.67 (95% CI, 0.53–0.84), which was associated with a 33% reduction in the risk of death. Patients diagnosed with MBC during earlier time periods showed poorer survival than those who were diagnosed during later time periods (2000–2004 vs. 2010–2013, *P* = 0.001) in univariate analysis; however, in multivariate analysis, there was no significant improvement in survival over time (Table 2).

**Table 2:** Univariate and multivariate analysis for overall survival.

Variable		Univariate		Multivariate	
		Hazard ratio	*P* value	Hazard ratio	*P* value
Age	≥ 40 years vs. < 40 years	0.94	0.570		
		(0.72–1.18)			
Disease status	MBC at initial diagnosis	1.34	0.155	1.30	0.214
	vs. DRFI ≥ 5 years	(0.89–2.02)		(0.86–1.95)	
	DRFI < 5 years vs. DRFI	2.64	<0.001	2.18	<0.001
	≥ 5 years	(1.96–3.57)		(1.60–2.98)	
Biological subtype	HER2+ vs. HR+	1.39	0.003	1.27	0.031
		(1.12–1.72)		(1.02–1.58)	
	TNBC vs. HR+	2.36	<0.001	1.94	<0.001
		(1.90–2.93)		(1.55–2.43)	
Time periods	2000–2004 vs. 2010–2013	1.65	0.001	1.18	0.276
		(1.24–2.20)		(0.87–1.59)	
	2005–2009 vs. 2010–2013	1.23	0.106	1.02	0.862
		(0.94–1.58)		(0.79–1.32)	
Clinical trial	No vs. Yes	1.50	0.001	1.32	0.021
enrollment		(1.19–1.88)		(1.04–1.67)	

Values in parentheses are 95% confidence intervals. MBC, metastatic breast cancer; DRFI, DRFI, distant recurrence-free interval.

### Subgroup analysis

**Fig 4:**
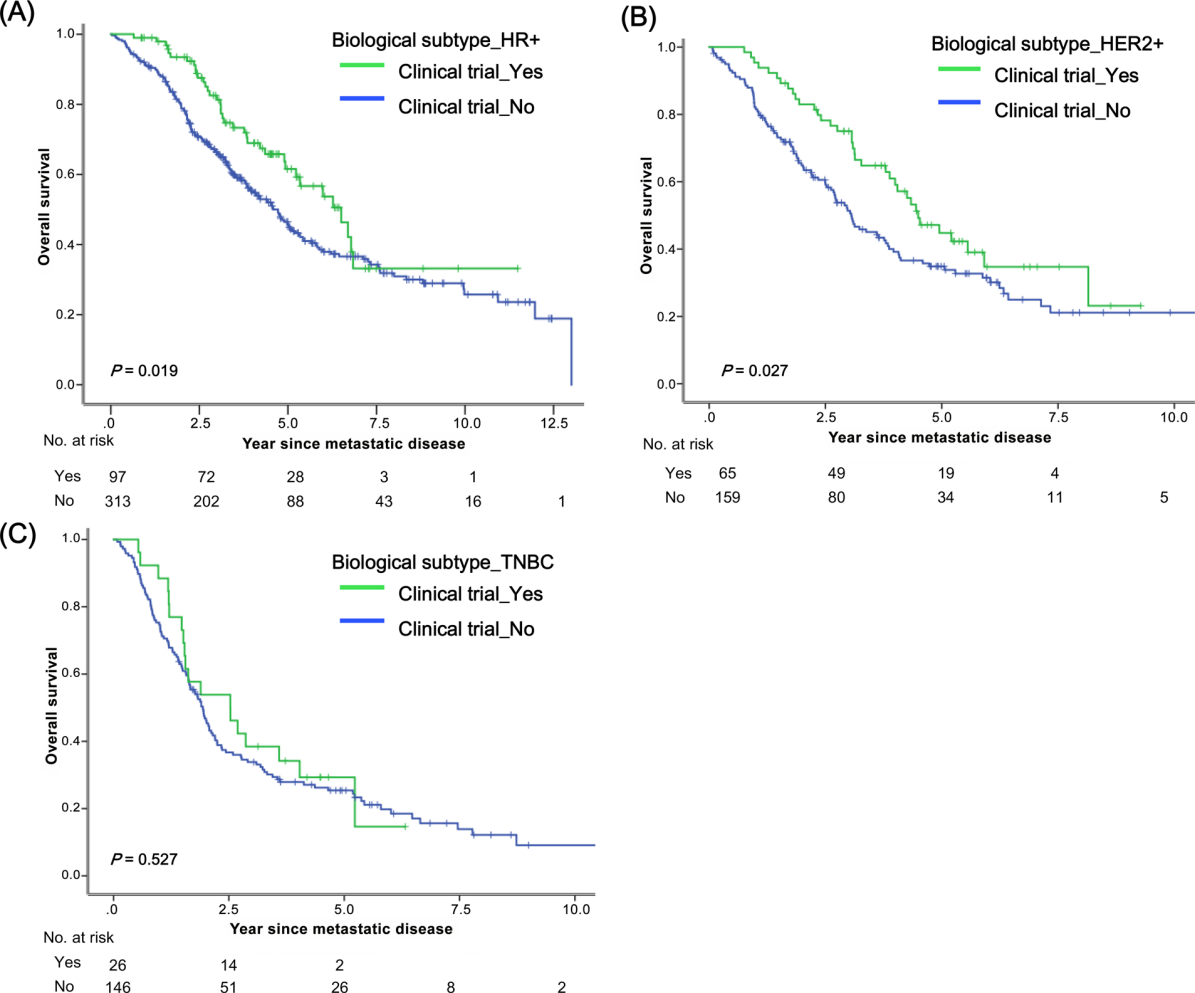
Survival after diagnosis with MBC according to clinical trial enrollment stratified by biologic subtype: (a) HR+, (b) HER2+, and (c) TNBC.

Fig 4 illustrates survival from the time of diagnosis with MBC according to clinical trial enrollment, stratified by biological subtype. Patients with biological subtype HR+ who were enrolled in clinical trials experienced survival benefits (*P* = 0.019, Fig 4A) as did those with subtype HER2+ (*P* = 0.027, Fig 4B); however, this was not true of TNBC (*P* = 0.527, Fig 4C). As shown in the S2 Fig, survival benefit was consistent across age subgroups. We noted significant improvements in overall survival associated with clinical trial enrollment among patients with certain biological subgroups (HR+ or HER2+), shorter DRFI (< 5 years), and certain time periods of recurrence (2010–2013).

## Discussion

Recent epidemiological data indicate significant worldwide decreases in breast cancer mortality over the past few decades [19, 20]. Much of this improvement in outcomes is attributed to earlier detection strategies, the greater use of adjuvant hormone and cytotoxic treatments, and more efficacious and numerous treatment options for a patients with MBC [9, 21–23]. The results of the present study provide further evidence of improvements in survival associated the better treatment integration of clinical trial.

Consistent with the results of previous studies [6, 7, 24–26], our analyses demonstrate that certain biologic subtypes (HER2+ or TNBC) and short DRFI (< 5 years) are associated with inferior survival. After adjusting for these variables, a trend toward better survival was seen among patients who were enrolled in clinical trials (*P* = 0.021). The impact of clinical trial enrollment on survival has not broadly translated into improved survival. Subgroup analysis showed that patients with biological subtype HER2+ and later time period of recurrence (2005–2009 or 2010–2013) experienced greater benefit from clinical trial enrollment. This improvement could be explained by the increased availability of effective drugs, especially new HER2-targeting agents. In the late 2000s and the early 2010s, several clinical trials for HER2-targeting agents beyond trastuzumab, including lapatinib [27], pertuzumab [28], and the antibody drug conjugate ado-trastuzumab emtansine or T-DM1 [29] were established. Furthermore, advances in understanding tumor biology, particularly signaling pathway, have changed the treatment paradigm for MBC [30]. The combination of the mTOR inhibitor everolimus with the aromatase inhibitor exemestane has increased efficacy in patients with ER+ MBC [31]. In the current study, the patients in later time periods had a more chance to use novel agents or new combination regimens. Patients with shorter DRFI (< 5 years) also experienced benefits from enrollment in clinical trials. This finding may reflect the fact that HER2+ disease is associated with early recurrence. It is noteworthy that patients with TNBC did not show any improvement in survival associated with clinical trial enrollment. This suggests that there remains a critical need for the development of therapies, especially those that focus on clinically efficient molecular targets, for patients with MBC.

Previous reports indicated that the period of diagnosis is an independent predictor of survival [6, 32, 33]. On the contrary, in the present study, we found that the survival of MBC patients has improved over time, but the time period of diagnosis with MBC was not related to prognosis after adjustment for multiple prognostic covariates. Several factors could mediate time-related bias: changes in radiologic facilities for detection and diagnosis, changes in diagnostic criteria, evolution of clinical characteristics, and improvement of supportive care. In the present study, the preponderance of favorable tumor biologies such as HR+, longer DRFI, and greater enrollment in clinical trials among patients who were diagnosed during later time periods might explain the improvement in survival over the time that we observed. Nevertheless, this improvement over time may be mainly due to new advances including the discovery of oncogenic drivers and therapeutic agents for these driver mutations.

The current study has a number of limitations. This was a retrospective single center cohort study and therefore, selection bias could be a confounding variable. Most important critical weakness in this study is that patients enrolled in clinical trials are a highly selected group, and are not necessarily representative of the general population of patients with MBC. We were unable to evaluate clinical findings, such as performance status and organ function, that may be associated with more favorable survival outcomes among patients who were enrolled in clinical trials.

Our findings, although not conclusive, suggest that breast cancer survival is improved by enrollment in clinical trials. The development of targeted, biologically-based therapies may be the most promising strategy for prolonging survival among MBC patients. Our results support the ethical background for conducting clinical trials.

### Clinical practice points

- The number of clinical trials exploring treatments for MBC that is refractory to conventional treatment has increased rapidly.
- The incorporation of clinical trials improved overall survival among patients with MBC.
- Patients with biologic subtype HR+ and HER2+ experienced survival benefits, however, this was not maintained in patients with triple negative breast cancer.
- Our finding suggest that development of targeted therapeutics available for everyday clinical practice should be needed for MBC patients.

## Supporting Information

S1 Fig Survival curves according to (a) disease status at diagnosis with MBC, (b) time period of diagnosis with MBC, and (c) time period of clinical trial enrollment. (TIF)

S2 Fig Subgroup analysis. (TIF)

S1 Table Chemotherapies and hormone therapies prescribed for the treatment of metastatic breast cancer for the time cohort. (DOCX)

S2 Table Clinical trials for metastatic breast cancer. (DOCX)
