# Supplementary figures and images for: The Impacts of Inclusion in Clinical Trials on Outcomes among Patients with Metastatic Breast Cancer (MBC)
Source: PLoS One. 2016 Feb 22;11(2):e0149432. doi: 10.1371/journal.pone.0149432 (PMC4763476; doi:10.1371/journal.pone.0149432)

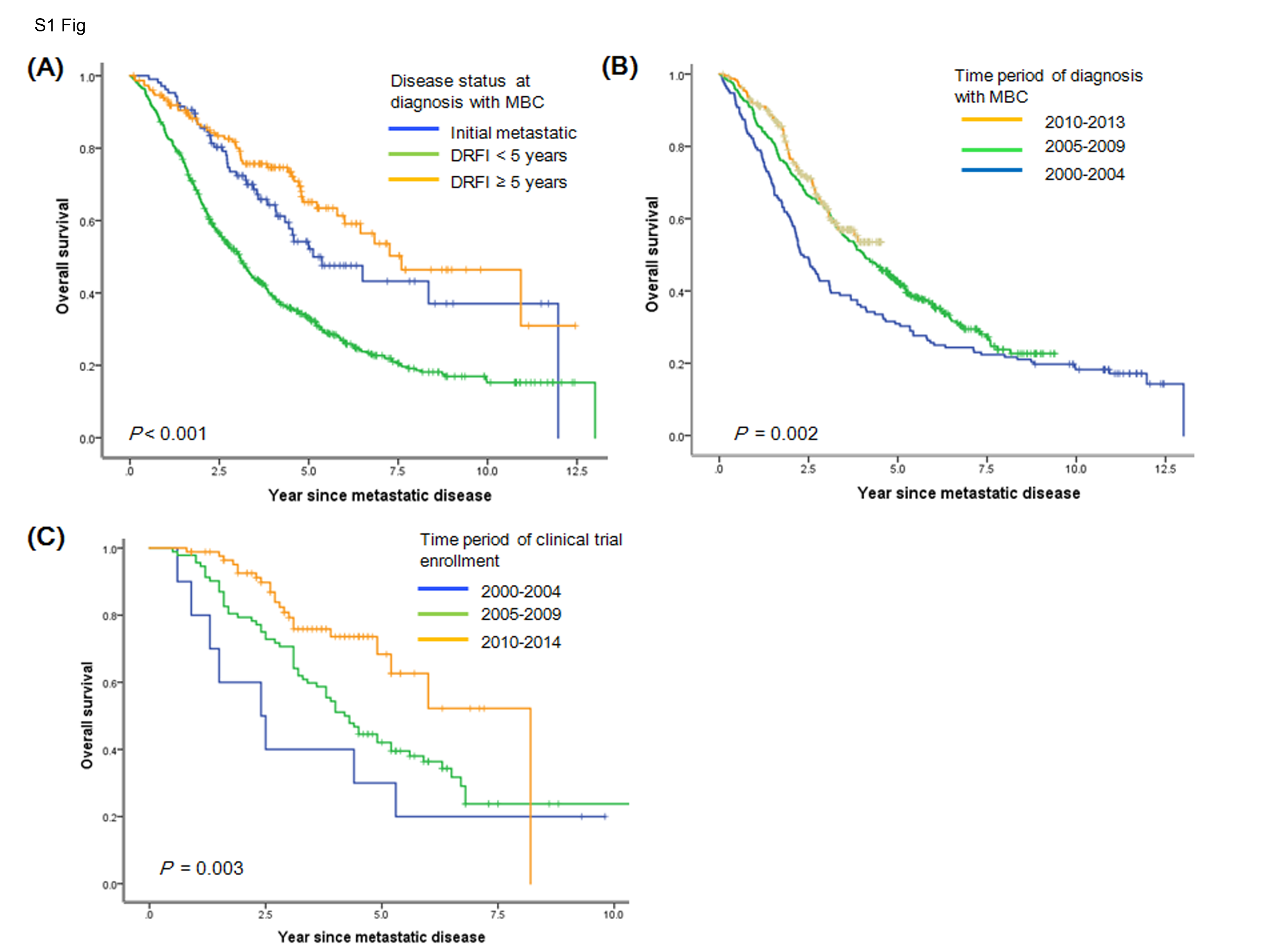

Supplement: S1 Fig — (TIF) [file pone.0149432.s001.tif]

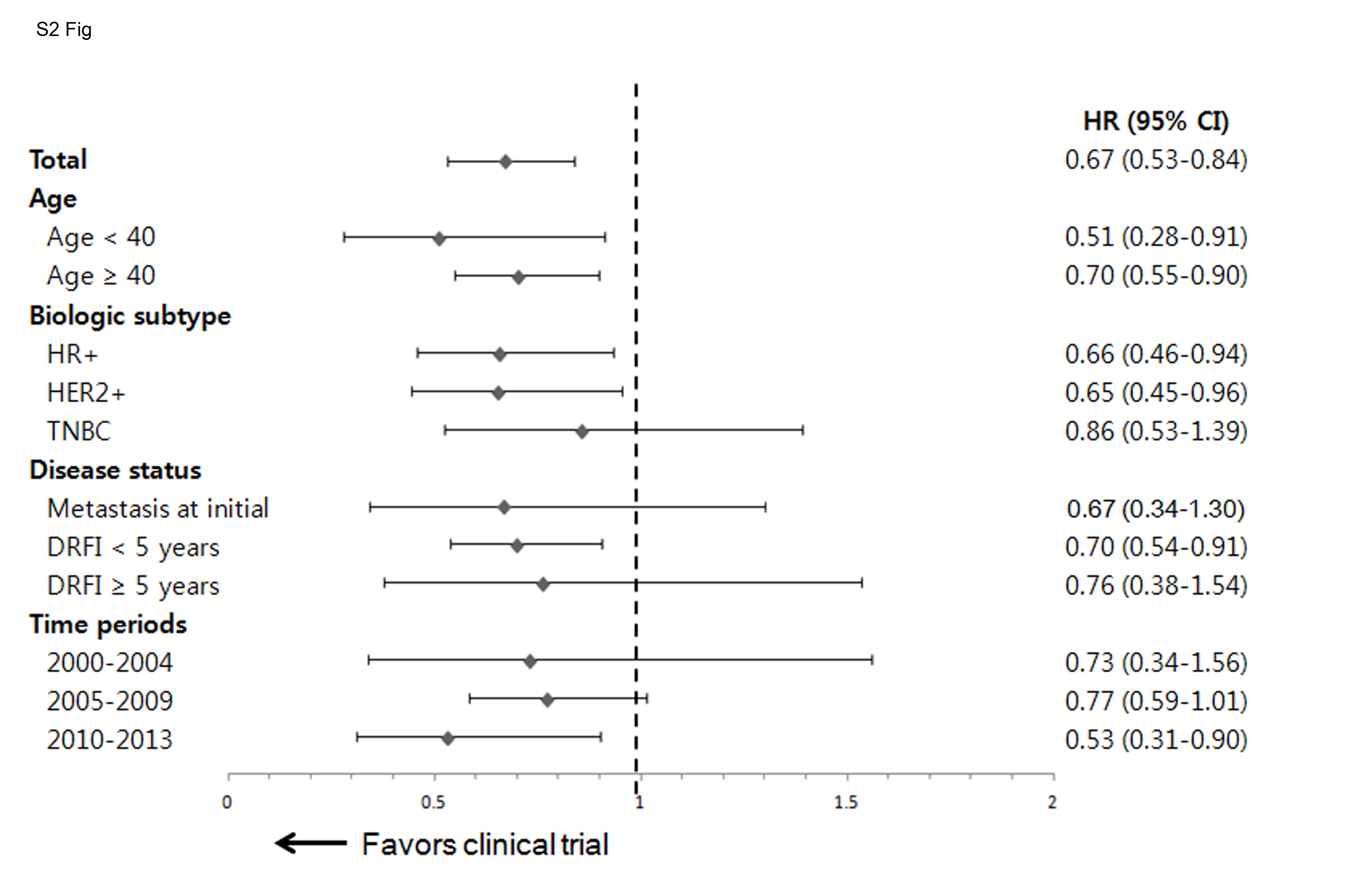

Supplement: S2 Fig — (TIF) [file pone.0149432.s002.tif]
