# Supplementary material for: The Impacts of Inclusion in Clinical Trials on Outcomes among Patients with Metastatic Breast Cancer (MBC)
Source: PLoS One. 2016 Feb 22;11(2):e0149432. doi: 10.1371/journal.pone.0149432 (PMC4763476; doi:10.1371/journal.pone.0149432)
Supplement: S1 Table — (DOCX) [file pone.0149432.s003.docx]

**S1 Table.** Chemotherapies and hormone therapies prescribed for the treatment of metastatic breast cancer for the time cohort

|  | **Therapy** | **Total**  **n = 806** | **2000-2004**  **n = 152** | **2005-2009**  **n = 415** | **2010-2013**  **n = 239** |
| --- | --- | --- | --- | --- | --- |
| Palliative cytotoxic chemotherapy | No | 154 (19) | 33 (22) | 75 (18) | 46 (19) |
|  | Yes | 652 (81) | 119 (78) | 340 (82) | 193 (81) |
| Chemotherapy  Yes (n = 652) | Anthracyclines† | 329 (51) | 68 (57) | 188 (55) | 73 (38) |
|  | Paclitaxel | 403 (62) | 71 (60) | 224 (66) | 108 (56) |
|  | Docetaxel | 366 (56) | 75 (63) | 191 (56) | 100 (52) |
|  | Vinorelbine† | 210 (32) | 45 (38) | 127 (37) | 38 (20) |
|  | Gemcitabine | 351 (54) | 64 (54) | 193 (57) | 94 (49) |
|  | Capecitabine | 504 (77) | 90 (76) | 289 (85) | 125 (65) |
|  | Eribulin† | 48 (7) | 6 (5) | 21 (6) | 21 (11) |
|  | Trastuzumab | 159 (24) | 16 (13) | 97 (29) | 46 (24) |
| Palliative hormone therapy | No | 397 (49) | 76 (50) | 210 (51) | 111 (46) |
|  | Yes | 409 (51) | 76 (50) | 205 (49) | 128 (54) |
| Hormone therapy  Yes (n = 409) | Anastrozole | 91 (22) | 29 (38) | 48 (23) | 14 (11) |
|  | Letrozole | 234 (57) | 34 (45) | 125 (61) | 75 (59) |
|  | Exemestane | 191 (47) | 28 (37) | 97 (47) | 66 (52) |
|  | Fulvestrant | 43 (11) | 8 (11) | 15 (7) | 20 (16) |
|  | Tamoxifen | 184 (45) | 31 (41) | 88 (43) | 65 (51) |
