# Supplementary material for: The Impacts of Inclusion in Clinical Trials on Outcomes among Patients with Metastatic Breast Cancer (MBC)
Source: PLoS One. 2016 Feb 22;11(2):e0149432. doi: 10.1371/journal.pone.0149432 (PMC4763476; doi:10.1371/journal.pone.0149432)
Supplement: S2 Table — (DOCX) [file pone.0149432.s004.docx]

**S2 Table.** Clinical trials for metastatic breast cancer

| **Clinical trial_ 2000-2004** |
| --- |
| EGF30008 study: A Randomized, Double-Blind, Placebo-Controlled, Multicenter, Phase III Study Comparing GW572016 and Letrozole Versus Letrozole in Subjects With Estrogen/Progesterone Receptor-Positive Advanced or Metastatic Breast Cancer |
| EGF30001 study: A Randomized, Multicenter, Double-Blind, Placebo-Controlled, 2-Arm, Phase III Study of Oral GW572016 in Combination With Paclitaxel in Subjects Previously Untreated or Advanced or Metastatic Breast Cancer |
| Palliative capecitabine study |
| **Clinical trial_ 2005-2009** |
| Paclitaxel & Gemcitabin Phase III study |
| AVADO study: Phase III Study of Bevacizumab Plus Docetaxel Compared With Placebo Plus Docetaxel for the First-Line Treatment of Human Epidermal Growth Factor Receptor 2–Negative Metastatic Breast Cancer |
| SoFEA study: A Partially-Blind Phase III Randomized Trial of Fulvestrant (Faslodex™) With or Without Concomitant Anastrozole (Arimidex™) Compared With Exemestane in Postmenopausal Women With ER+ve Locally Advanced/Metastatic Breast Cancer Following Progression on Non-Steroidal Aromatase Inhibitors |
| EGF 103659 study: An Open-Label Expanded Access Study of Lapatinib and Capecitabine Therapy in Subjects With ErbB2 Overexpressing Locally Advanced or Metastatic Breast Cancer |
| Fulvestrant study in metastatic breast cancer |
| BMS247550 study: Ixabepilone **(**BMS-247550**)** In Patients With Metastatic Breast Cancer |
| RPR109881 study: A Phase II Multi-Center, Open-Label, Non-Randomized Study of Intravenous RPR109881 Given Every 3 Weeks in Patients With Metastatic Breast Cancer Progressing After Therapy With Anthracyclines, Taxanes and Capecitabine |
| S188 study: A Randomized Trial of Gemcitabine Plus Docetaxel vs. Docetaxel Plus Capecitabine in Metastatic Breast Cancer in 1st and 2nd |
| CLEOPATRA study: A Phase III, Randomized, Double-blind, Placebo-controlled Clinical Trial to Evaluate the Efficacy and Safety of Pertuzumab + Trastuzumab + Docetaxel vs. Placebo + Trastuzumab + Docetaxel in Previously Untreated HER2-positive Metastatic Breast Cancer |
| TSU-68 study: A multicenter phase II study of TSU-68, a novel oral multiple tyrosine kinase inhibitor, in patients with metastatic breast cancer progressing despite prior treatment with an anthracycline-containing regimen and taxane |
| Ixabepilone study: A Randomized Phase III Trial of Ixabepilone Plus Capecitabine Versus Capecitabine in Patients With Metastatic Breast Cancer Previously Treated With an Anthracycline and a Taxane |
| THERESA study : T-DM1 vs TPC in HER-2(+) breast cancer, randomized, phase III |
| HKI-3003 study: Study Evaluating Neratinib Versus Lapatinib Plus Capecitabine For ErbB2 Positive Advanced Breast Cancer |
| EGF111767 study: An Open-Label Phase Ib Continuation Study of Lapatinib Monotherapy or Lapatinib in Combination with Other Anti-Cancer Treatment in Patients with Solid Tumors |
| TORCH study: A Phase II Trial of Oxaliplatin in Combination With S-1(SOX) in Patients With Recurrent or Metastatic Breast Cancer (MBC) Previously Treated With or Resistant to an Anthracycline and Taxane |
| EMILIA study: A phase III study of trastuzumab emtansine (T-DM1) versus capecitabine and lapatinib in HER2-positive locally advanced or metastatic breast cancer previously treated with trastuzumab and a taxane |
| **Clinical trial_2010-2014** |
| THERESA study: A Phase II Study of Vinorelbine Plus Trastuzumab in HER2 Overexpressing Metastatic Breast Cancer Pretreated with Anthracyclines and Taxanes |
| BEACON study: A Phase 3 Open-Label, Randomized, Multicenter Study of NKTR-102 Versus Treatment of Physician's Choice (TPC) in Patients With Locally Recurrent or Metastatic Breast Cancer Previously Treated With an Anthracycline, a Taxane and Capecitabine |
| LV study: Lapatinib+Vinorelbine vs vinorelbine] in HER2(+) breast cancer |
| EFG114299 study: A Phase III trial to compare the safety and efficacy of lapatinib plus trastuzumab plus an aromatase inhibitor (AI) vs. trastuzumab plus an AI vs. lapatinib plus an AI as 1st- or 2nd- line therapy in postmenopausal subjects with hormone receptor+, HER2+ metastatic breast cancer (MBC) who received prior trastuzumab and endocrine therapies |
| FLAG study: A Randomized Phase II Study OF Goserelin (G) Plus Fulvestrant (F) vs. G Plus Anastrozole (A)vs. G Alone for HR+, Tamoxifen Pretreated, Premenopausal Woman |
| ERIBULIN study: Eribulin in advanced/metastatic breast ca, phase IV |
| MARIANNE study: A Study of Trastuzumab-DM1 Plus Pertuzumab Versus Trastuzumab [Herceptin] Plus a Taxane in Patients With Metastatic Breast Cancer |
| EVEREXES study: A phase IIIb, multi-center, open-label, expanded access study of EVERolimus (RAD001) in combination with EXemestane in post-menopausal women with EStrogen receptor positive, human epidermal growth factor receptor 2 negative locally advanced or metastatic breast cancer (EVEREXES) |
| Paloma-3 study: Multicenter, Randomized, Double-blind, Placebo-controlled, Phase 3 Trial Of Fulvestrant (Faslodex (Registered)). With Or Without Pd-0332991 (Palbociclib) +/- Goserelin In Women With Hormone Receptor-positive, Her2-negative Metastatic Breast Cancer Whose Disease Progressed After Prior Endocrine Therapy |
| EMILIA study: A phase III study of trastuzumab emtansine (T-DM1) versus capecitabine and lapatinib in HER2-positive locally advanced or metastatic breast cancer previously treated with trastuzumab and a taxane |
| CLEOPATRA study: A Phase III, Randomized, Double-blind, Placebo-controlled Clinical Trial to Evaluate the Efficacy and Safety of Pertuzumab + Trastuzumab + Docetaxel vs. Placebo + Trastuzumab + Docetaxel in Previously Untreated HER2-positive Metastatic Breast Cancer |
| BIBW study: Single-arm, Open-label, Multicentre Phase II Study Evaluating the Efficacy and Safety of BIBW 2992 (Afatinib) in Combination With Vinorelbine for the Treatment of Patients With Metastatic Breast Cancer |
| CT-P6 study: Double-blind, Randomised, Parallel Group, Phase III Study |
| HKI-3003 study :Neratinib: |
| HKI3005 study: Pallitive Paclitaxel + Neratinib |
| Paclitaxel & Gemcitabin Phase III study |
